# Characteristics and Treatment Outcomes of Patients with MDR and XDR Tuberculosis in a TB Referral Hospital in Beijing: A 13-Year Experience

**DOI:** 10.1371/journal.pone.0019399

**Published:** 2011-04-29

**Authors:** Cui Hua Liu, Ling Li, Zhi Chen, Qi Wang, Yong Liang Hu, Baoli Zhu, Patrick C. Y. Woo

**Affiliations:** 1 CAS Key Laboratory of Pathogenic Microbiology and Immunology, Institute of Microbiology, Chinese Academy Sciences, Beijing, China; 2 Institute for Tuberculosis Research, the 309 Hospital, Beijing, China; 3 Institute of Genetic Engineering, Southern Medical University, Guangzhou, China; 4 State Key Laboratory of Emerging Infectious Diseases, The University of Hong Kong, Hong Kong, Special Administrative Region, People's Republic of China; University of Cape Town, South Africa

## Abstract

**Background:**

Information on treatment outcomes among hospitalized patients with multidrug-resistant tuberculosis (MDR-TB) and extensively drug-resistant tuberculosis (XDR-TB) are scarce in China.

**Methodology/Principal Findings:**

We conducted this retrospective study to analyze the characteristics and treatment outcomes in MDR- and XDR-TB patients in the 309 Hospital in Beijing, China during 1996–2009. Socio-demographic and clinical data were retrieved from medical records and analyzed. Logistic regression analysis was performed to identify risk factors associated with poor treatment outcomes and Cox proportional hazards regression model was further used to determine risk factors associated with death in TB patients. Among the 3,551 non-repetitive hospitalized TB patients who had drug susceptibility testing (DST) results, 716 (20.2%) had MDR-TB and 51 (1.4%) had XDR-TB. A total of 3,270 patients who had medical records available were used for further analyses. Treatment success rates (cured and treatment completed) were 90.9%, 53.4% and 29.2% for patients with non-MDR-TB, patients with MDR-TB excluding XDR-TB and patients with XDR-TB, respectively. Independent risk factors associated with poor treatment outcomes in MDR-TB patients included being a migrant (adjusted OR = 1.77), smear-positivity at treatment onset (adjusted OR = 1.94) and not receiving 3 or more potentially effective drugs (adjusted OR = 3.87). Independent risk factors associated with poor treatment outcomes in XDR-TB patients were smear-positivity at treatment onset (adjusted OR = 10.42) and not receiving 3 or more potentially effective drugs (adjusted OR = 14.90). The independent risk factors associated with death in TB patients were having chronic obstructive pulmonary disease (adjusted HR = 5.25) and having hypertension (adjusted HR = 4.31).

**Conclusions/Significance:**

While overall satisfactory treatment success for non-MDR-TB patients was achieved, more intensive efforts should be made to better manage MDR- and XDR-TB cases in order to improve their treatment outcomes and to minimize further emergence of so-called totally drug-resistant TB cases.

## Introduction

Although significant achievements have been made in controlling TB in China over the last decade [1], [2], multidrug-resistant (MDR-) and extensively drug-resistant tuberculosis (XDR-TB) cases have been widespread, causing a public health problem in China and worldwide in recent years [3]–[8]. According to the 2010 WHO report on drug resistance surveillance [8], the estimated number of MDR-TB cases has reached 440,000 globally in 2008 with nearly 50% of the cases coming from India and China. By January 2010, 58 countries had reported at least one case of XDR-TB. The number of deaths caused by MDR-TB was estimated to be 150,000 in 2008. The latest nationwide baseline survey for TB drug resistance in China from 2007 to 2008 showed that 8.32% of pulmonary TB patients in China suffered from MDR-TB and 0.68% from XDR-TB [8].

The treatment of patients with MDR- and XDR-TB is more complex, toxic and costly and less effective than treatment for other forms of TB. Also, the treatment outcomes of patients with MDR- and XDR-TB are greatly variable according to the different settings and regions of the world [9]–[20]. A better understanding of risk factors associated with poor treatment outcomes among MDR- and XDR-TB patients would be useful to provide better case management. Such data among hospitalized TB patients in China are lacking. We performed a retrospective study to determine the characteristics, treatment outcomes and risk factors associated with poor treatment outcomes among patients who were treated for MDR- and XDR-TB from 1996 to 2009 in the 309 Hospital in Beijing, China.

## Methods

### Ethics statement

All of the investigation protocols in this study were approved by the institutional ethics committee of the 309 Hospital, Beijing, China. Since it was a retrospective study, all the information of patients was routinely collected and recorded by attending physicians. Written consent was not required for this study since only routinely collected information was used, and confidentiality of clinical and laboratory information of the patients was maintained. Nevertheless, we obtained written informed consent from patients since the project started in 2009 to provide information for scientific studies. Permission for using the information in the medical records of the patients for research purposes was obtained from the 309 Hospital. The Institute ethics committee of the 309 Hospital reviewed that relevant ethical issues in this study were well considered.

### Study subjects and data collection

Beijing includes 18 districts, covering a total area of 16,800 km^2^. In 2006, the region held 11,976,900 permanent residents and 5,475,000 migrants from other provinces in China [21]. The 309 Hospital, which has a 244-bed TB centre, is the only TB referral hospital in the urban area of Beijing. The patients treated in the 309 Hospital are either self-referred or referred here by the clinicians from the general hospitals, community clinics, as well as district TB prevention and treatment clinics in Beijing and other provinces in China.

Drug susceptibility testing (DST) results and medical records of all TB patients receiving inpatient treatment in our hospital between July 1, 1996 and July 1, 2009 were retrospectively reviewed. All hospitalised TB patients with both DST results and medical records available were included for further analysis. Basic socio-demographic and clinical characteristics were obtained from the medical records. Treatment outcomes during the period 1996–2009 were determined by reviewing the medical records from the in-patient treatment and out-patient treatment, as well as from the telephone follow-up protocols. All study patients except for those who defaulted or were transferred out were monitored for further deaths for 2 years after the start of the treatment.

### *M. tuberculosis* cultures and drug susceptibility testing

Specimens were routinely collected from confirmed or suspected TB cases. Acid-fast bacilli (AFB) smear microscopy was assessed by Ziehl-Neelsen staining. Specimens were cultured using the BACTEC 960 system (Becton Dickinson Diagnostic Systems, Sparks, MD, USA) according to the manufacturer's instructions. Identification of *M. tuberculosis* was performed using p-nitrobenzoic acid and thiophene carboxylic acid hydrazine resistance tests as well as PCR tests. DST was performed using the proportion method and Löwenstein-Jensen medium. The concentrations of the drugs used were as follows: isoniazid (1 mg/L), rifampin (50 mg/L), ethambutol (5 mg/L), streptomycin (10 mg/L), pyrazinamide (50 mg/L), ofloxacin (2 mg/L), levofloxacin (2 mg/L), kanamycin (10 mg/L), para-aminosalicylic acid (1 mg/L). Quality control was routinely performed during DST using the reference strains provided by the National Institute for the Control of Pharmaceutical and Biological Products (China). Periodic external quality assessment of the performance of DST results was conducted by the Tuberculosis Reference Laboratory (TRL) at the Beijing Research Institute for Tuberculosis Control. All drugs were obtained from Sigma Life Science Company (USA).

### Definitions

New TB cases were defined as TB patients who had denied having had any prior anti-TB treatment or who received anti-TB treatment for 30 days or less. Retreatment TB cases were TB patients who had been receiving TB treatment for at least 30 days or who had had documented evidence of prior treatment from the case report or surveillance database [22]. MDR-TB was defined as TB with resistance to at least isoniazid and rifampin. XDR-TB was defined as TB with resistance to at least isoniazid, rifampin, a fluoroquinolone (e.g. ofloxacin, levofloxacin) and one of three injectable second-line drugs (capreomycin, kanamycin, and amikacin) [22], [23]. Mono-resistant TB was defined as resistance to a single first-line anti-TB drug. Poly-resistant TB was defined as resistance to two or more of the first-line anti-TB drugs, but not to both isoniazid and rifampin [22]. In this study, non-MDR-TB cases are all drug-susceptible, mono- and poly-resistant cases and excluding all MDR- and XDR-TB cases. Migrants and residents were defined as in a previous study [24]. Briefly, migrants were defined as individuals from other areas of China who moved to Beijing. Residents were defined as individuals who had a registered permanent residence in Beijing. Treatment outcomes were defined according to the WHO and International Union Against Tuberculosis and Lung Disease (IUATLD) guidelines with minor modifications [22], [25]. Cured was defined as a patient who had completed treatment according to program protocol and had been consistently culture-negative (with at least five results) for the final 12 months of treatment for TB. Definition of completed treatment was defined as a patient who had completed treatment according to program protocol but does not meet the definition for cured because of lack of bacteriological results. Died was defined as a patient who died for any reason during the course of TB treatment. Treatment was considered to have failed if two or more of the five cultures recorded in the final 12 months of therapy were positive, or if any one of the final three cultures was positive. Defaulted was defined as a patient whose TB treatment was interrupted for 2 or more consecutive months for any reason. Transferred out was defined as a patient who had been transferred to another reporting and recording unit and for whom the treatment outcome was unknown. Cured and treatment completed were combined as treatment success, whereas others were combined as poor treatment outcomes.

### Statistical analysis

All data were analysed using the SPSS software (15.0 version). Comparisons of categorical variables were performed using the Pearson Chi-square test to compare different groups. Univariate and multivariate logistic regression analyses were performed to evaluate the association between patient characteristics and poor treatment outcomes. Cox proportional hazards regression model was used to determine factors associated with risk of death in TB patients. All variables with a *P* value<0.20 in the univariate analysis were included in the multivariate logistic regression model. A *P* value of <0.05 was considered to be statistically significant.

## Results

### General characteristics of the study population

**Figure 1:**
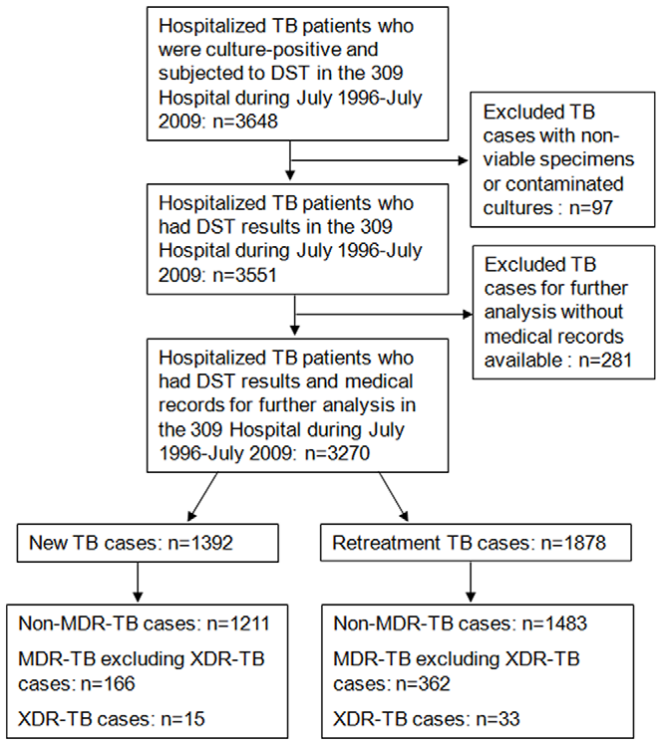
Study population of hospitalized TB cases in the 309 Hospital, July 1996–July 2009. TB = tuberculosis. MDR = multidrug-resistant. XDR = extensively drug-resistant.

From July 1996 to July 2009, 5201 TB patients including 2181 (41.9%) new cases and 3020 (58.1%) retreatment cases received in-patient treatment for TB in the TB center of the 309 Hospital. Of these, 3648 (70.1%) patients were culture positive for *M. tuberculosis* and were subjected to DST. Ninety-seven patients (2.7%) were further excluded as a result of non-viable specimens or contaminated cultures. Of the remaining 3551 patients, 716 (20.2%) had MDR-TB and 51 (1.4%) had XDR-TB. The final analysis included 3270 patients who had medical records available (Figure 1). The patients were classified into three groups: non-MDR-TB (n = 2694), MDR-TB excluding XDR-TB (n = 528), and XDR-TB (n = 48). Among the 2694 patients in the non-MDR-TB group, 1321 (49.0%) had drug-susceptible TB, 558 (20.7%) had mono-resistant TB, 663 (24.6%) had poly-resistant TB and 152 (5.6%) had TB that was resistant to second-line anti-TB drugs only. We compared the characteristics including sex, age and treatment history for patients who were included and excluded from the study population and found no significant differences between them (data not shown). Although 3 out of the 5201 TB patients tested positive for HIV, all of the 3270 patients who were included for final analysis were tested negative for HIV. The median age of the patients was 41.0 years (range 2–99). Most patients were male (70.1%), aged 15–44 years (56.8%), had pulmonary TB (92.2%), were smear-positive at treatment onset (73.0%), had cavitary diseases (54.4%), and were retreatment cases (57.4%). There were 1421 (43.5%) patients who were Beijing residents and 1849 (56.5%) migrants from other provinces in China. We observed a significantly higher percentage of retreatment cases, smear-positive cases, as well as cases with 4 or more years of TB disease among patients with MDR-TB and XDR-TB compared to non-MDR-TB patients. There were a much higher proportion of patients with cavitary disease, chronic obstructive pulmonary disease, and abnormal liver function among the patients with XDR-TB compared to patients in the other two groups. More detailed information on socio-demographic and clinical patient characteristics are shown in Table 1 and Table 2, respectively.

**Table 1:** Socio-demographic characteristics of hospitalized TB patients in the 309 Hospital.

Characteristics	Total	Patients with non-MDR -TB	Patients with MDR-TB excluding XDR-TB	Patients with XDR-TB	P
	n = 3270(%)	n = 2694 (%)	n = 528 (%)	n = 48 (%)	
Gender					0.028
Male	2292 (70.1)	1911 (70.9)	345 (65.3)	36 (75.0)	
Female	978 (29.9)	783 (29.1)	183 (34.7)	12 (25.0)	
Age					<0.001
0–14	18 (0.6)	12 (0.4)	4 (0.8)	2 (4.2)	
15–29	974 (29.8)	809 (30.0)	149 (28.2)	16 (33.3)	
30–44	884 (27.0)	696 (25.8)	171 (32.4)	17 (35.4)	
45–59	668 (20.4)	587 (21.8)	79 (15.0)	2 (4.2)	
60–74	451 (13.8)	356 (13.2)	87 (16.5)	8 (16.7)	
75–	275 (8.4)	234 (8.7)	38 (7.2)	3 (6.2)	
Marital status					0.023
Married	2443 (74.7)	2000 (74.2)	413 (78.2)	30 (62.5)	
Single	827 (25.3)	694 (25.8)	115 (21.8)	18 (37.5)	
Residence situation					0.143
Beijing Resident	1421 (43.5)	1192 (44.2)	210 (39.8)	19 (39.6)	
Migrant	1849 (56.5)	1502 (55.8)	318 (60.2)	29 (60.4)	
Living area					0.654
Rural area	1172 (35.8)	961 (35.7)	196 (37.1)	15 (31.2)	
Urban area	2098 (64.2)	1733 (64.3)	332 (62.9)	33 (68.8)	
Ethnicity					0.571
The largest group (Han)	3153 (96.4)	2600 (96.5)	508 (96.2)	45 (93.8)	
Ethnic groups	117 (3.6)	94 (3.5)	20 (3.8)	3 (6.2)	
Smoking, yes (n = 3119)	526 (16.9)	443 (17.2)	78 (15.7)	5 (11.9)	0.506
Alcohol abuse, yes (n = 3151)	142 (4.5)	120 (4.6)	20 (4.0)	2 (4.5)	0.857

TB = tuberculosis;

MDR = multidrug-resistant;

XDR = extensively drug-resistant.

**Table 2:** Clinical characteristics of hospitalized TB patients in the 309 Hospital.

Characteristics	Total	Patients with non-MDR -TB	Patients with MDR-TB excluding XDR-TB	Patients with XDR-TB	P
	n = 3270(%)	n = 2694 (%)	n = 528 (%)	n = 48 (%)	
Sites of TB					0.013
Extrapulmonary TB	256 (7.8)	228 (8.5)	25 (4.7)	3 (6.2)	
Pulmonary TB	3014 (92.2)	2466 (91.5)	503 (95.3)	45 (93.8)	
Lower lung field TB (n = 3024)	1575 (52.1)	1286 (52.0)	262 (52.0)	27 (60.0)	0.563
TB history					<0.001
New	1392 (42.6)	1211 (45.0)	166 (31.4)	15 (31.2)	
Retreatment	1878 (57.4)	1483 (55.0)	362 (68.6)	33 (68.8)	
Smear-positivity at treatment onset (n = 2674)	1951 (73.0)	1526 (70.9)	392 (82.2)	33 (75.0)	<0.001
Radiological findings at onset					0.545
Non-cavitary	1492 (45.6)	1239 (46.0)	234 (44.3)	19 (39.6)	
Cavitary disease	1778 (54.4)	1455 (54.0)	294 (55.7)	29 (60.4)	
Family history of TB, yes (n = 3205)	133 (4.1)	100 (3.8)	31(6.2)	2 (4.3)	0.046
Underlying diseases					
Diabetes mellitus	393 (12.0)	331 (12.3)	59 (11.2)	3 (6.2)	0.359
Chronic obstructive pulmonary disease	168 (5.1)	137 (5.1)	22 (4.2)	9 (18.8)	<0.001
Abnormal liver function	190 (5.8)	171 (6.3)	8 (1.5)	11 (22.9)	<0.001
Hepatitis	112 (3.4)	90 (3.3)	19 (3.6)	3 (6.2)	0.532
Malignancy	25 (0.8)	24 (0.9)	1 (0.2)	0 (0.0)	0.198
Hypertension	172 (5.3)	145 (5.4)	22 (4.2)	5 (10.4)	0.142
4 or more previous hospitalization for TB	175 (5.4)	135 (5.0)	36 (6.8)	4 (8.3)	0.157
4 or more years of TB disease	1720 (52.6)	1272 (47.2)	406 (76.9)	42 (87.5)	<0.001

TB = tuberculosis;

MDR = multidrug-resistant;

XDR = extensively drug-resistant.

### Anti-TB drug susceptibility patterns of the TB patients at treatment initiation

At treatment initiation, 8.7% (46/528) of MDR-TB excluding XDR-TB patients and 18.8% (9/48) of patients with XDR-TB showed resistance to all first-line anti-TB drugs. The percentage of patients with resistance to 5 or more anti-TB drugs was 23.3% (629/2694), 46.8% (247/528), and 89.6% (43/48), for patients with non-MDR-TB, patients with MDR-TB excluding XDR-TB, and patients with XDR-TB respectively. Patients with non-MDR-TB had median resistance to 3 (range 0–6) anti-TB drugs. Patients with MDR-TB excluding XDR-TB had median resistance to 4 (range 2–9) anti-TB drugs, whereas those with XDR-TB had median resistance to 6 (range 4–9) drugs. More detailed information on patient drug susceptibility pattern is shown in Table 3.

**Table 3:** Anti-TB drugs used in treatment regimens for hospitalized TB patients in the 309 Hospital.

	Patients with non-MDR -TB (n = 2694)			Patients with MDR-TB excluding XDR-TB (n = 528)			Patients with XDR-TB (n = 48)		
	Patients given drug	Treatment duration (months; median, IQR)	Resistance pattern	Patients given drug	Treatment duration (months; median, IQR)	Resistance pattern	Patients given drug	Treatment duration (months; median, IQR)	Resistance pattern
isoniazid	2031 (75.4%)	5.1 (3.0–7.8)	752 (27.9%)	245 (46.4%)	2.2 (1.5–2.9)	528 (100.0%)	28 (58.3%)	2.1 (0.5–3.0)	48 (100.0%)
rifampin	1937 (71.9%)	5.9 (2.3–8.2)	430 (16.0%)	259 (49.1%)	2.3 (1.0–2.6)	528 (100.0%)	26 (54.2%)	2.4 (0.5–3.3)	48 (100.0%)
streptomycin	499 (18.5%)	1.8 (1.0–2.9)	920 (34.1%)	85 (16.1%)	2.2 (1.5–3.0)	274 (51.9%)	13 (27.1%)	2.8 (1.1–3.0)	24 (50.0%)
ethambutol	1644 (61.0%)	5.1 (3.3–6.2)	1004 (37.3%)	326 (61.7%)	6.7 (4.1–14.8)	278 (52.6%)	31 (64.6%)	6.9 (5.8–17.5)	23 (47.9%)
pyrazinamide	1396 (51.8%)	2.6 (2.0–4.1)	259 (9.6%)	457 (86.6%)	5.7 (3.6–8.3)	144 (27.3%)	41 (85.4%)	5.1 (3.1–9.6)	15 (31.3%)
ofloxacin	101 (3.7%)	6.0 (2.1–9.2)	200 (7.4%)	84 (15.9%)	9.3 (6.0–18.6)	66 (12.5%)	4 (8.3%)	4.1 (3.3–6.5)	48 (100.0%)
levofloxacin	1427 (53.0%)	8.1 (4.1–15.8)	200 (7.4%)	218 (41.3%)	10.1 (4.0–18.3)	66 (12.5%)	22 (45.8%)	9.5 (3.8–17.3)	48 (100.0%)
kanamycin	237 (8.8%)	2.5 (1.6–4.3)	349 (13.0%)	89 (16.9%)	3.2 (2.1–8.5)	137 (25.9%)	10 (20.8%)	3.2 (0.5–8.4)	48 (100.0%)
para-aminosalicylic acid	391 (14.5%)	3.1 (2.0–5.6)	650 (24.1%)	265 (50.2%)	6.7 (2.7–7.8)	129 (24.4%)	30 (62.5%)	7.6 (3.1–6.8)	14 (29.2%)
amikacin	187 (6.9%)	4.6 (2.5–9.6)	ND	67 (12.7%)	5.9 (3.1–15.6)	ND	5 (10.4%)	5.7 (2.0–11.5)	ND
capreomycin	0	0	ND	6 (1.1%)	4.0 (2.9–8.7)	ND	0	0	ND
Moxifloxacin	0	0	ND	6 (1.1%)	3.1 (2.5–5.5)	ND	20 (41.7%)	5.0 (3.7–6.0)	ND
Ciprofloxacin	0	0	ND	3 (0.6%)	2.7 (2.0–5.7)	ND	0	0	ND
Thiacetazone	1307 (48.5%)	5.2 (3.7–14.1)	ND	284 (53.8%)	8.2 (4.3–16.1)	ND	29 (60.4%)	8.1 (5.6–16.8)	ND
Rifapentin	0	0	ND	225 (42.6%)	2.6 (1.5–4.5)	ND	20 (41.7%)	1.8 (1.3–5.9)	ND
Rifabutin	0	0	ND	3 (0.6%)	2.4 (1.4–5.1)	ND	4 (8.3%)	3.4 (2.0–5.6)	ND

TB = tuberculosis;

MDR = multidrug-resistant;

XDR = extensively drug-resistant.

### Anti-TB treatment regimens

There were no established standard guidelines for treating TB patients in the 309 Hospital before 2001. Since 2001, patients with culture-confirmed TB were treated according to the National Tuberculosis Programme (NTP) treatment guidelines. In general, new pulmonary TB patients received 2 months of treatment with streptomycin (S) or ethambutol (E), isoniazid (H), rifampin (R), and pyrazinamide (Z) during the intensive phase and 4 months of isoniazid and rifampin during the continuation phase (2S(E)HRZ/4HR). Retreatment cases received 3 months of intensive treatment and 5 months of continuation treatment according to the following regimen: 2SHRZE/1HRZE/5HRE. During the study period, there was no standard treatment strategy to guide the treatment of MDR-TB patients in the 309 Hospital. In general, at least 3 months of amikacin (AMK) or capreomycin (CPM), thiacetazone (TH), pyrazinamide, and ofloxacin (OFLX) were prescribed for the intensive phase, whilst TH and OFLX were prescribed for the continuation phase. The most recent DST results available suggest that other drugs such as levofloxacin, moxifloxacin, ciprofloxacin, and 2 modified forms of rifampin (rifapentine and rifabutin) are also useful in different combinations. Capreomycin, moxifloxacin, and rifabutin were introduced into the treatment regimen in 2000, 2005, and 2008, respectively. The remaining drugs that are listed in Table 3 have been available since 1996. Although the continuation treatment phase for confirmed MDR-TB cases has been recommended to be at least 18 months, individualised regimens were administered based mainly on each individual patient's physical and financial situation as well as DST results. The 3270 study subjects in our study were hospitalised for a median (range) duration of 213 days (1–605 days). For patients in the non-MDR-TB group, the median (IQR) in-patient treatment duration was 6.9 months (3.7–15.5 months), and the median (IQR) total treatment duration (including in-patient treatment and out-patient treatment) was 13.6 months (12.5–17.6 months). For patients in the MDR excluding XDR-TB group, the median (IQR) in-patient treatment duration was 8.6 months (4.1–17.5 months), and the median (IQR) total treatment duration was 21.9 months (21.1–22.6 months). For patients in the XDR-TB group, the median (IQR) in-patient treatment duration was 8.9 months (4.5–18.3 months), and median (IQR) total treatment duration was 22.3 months (20.6–23.4 months). Detailed information on the drugs used in the treatment regimens, as well as treatment durations for the study subjects, are shown in Table 3. In general, patients that had tested negative for any serious underlying diseases received in-patient treatment during intensive treatment phase, with out-patient treatment and telephone follow-up being received during the continuation treatment phase. The test for sputum-smears and/or cultures was conducted on a monthly basis.

### Treatment outcomes of TB patients

A treatment success rate of 84.0% was obtained for the study population. In comparison with patients with non-MDR-TB (90.9%), a much lower percentage of MDR-TB patients has successful treatment, which is 53.4% without XDR-TB and 29.2% with XDR-TB (Table 4). We also observed a higher percentage of treatment success for new cases than retreatment cases (Table 5), and for patients who received treatment after 2001 than before 2001 (Table 5).

**Table 4:** Comparison of treatment outcomes for hospitalized TB patients in the 309 Hospital.

Treatment outcome	Patients with non-MDR -TB	Patients with MDR-TB excluding XDR-TB	Patients with XDR-TB	Total
	n = 2694 (%)	n = 528 (%)	n = 48 (%)	n = 3270 (%)
Treatment success	2450 (90.9)	282 (53.4)	14(29.2)	2746(84.0)
Cured	1591 (59.0)	170 (32.2)	5 (10.4)	1766 (54.0)
Treatment completed	859 (31.9)	112 (21.2)	9 (18.8)	980 (30.0)
Poor treatment outcomes	244 (9.1)	246 (46.6)	34 (70.8)	524 (16.0)
Died	31 (1.2)	16 (3.0)	3 (6.3)	50 (1.5)
Failed	132 (4.9)	131 (24.8)	21 (43.8)	284 (8.7)
Defaulted	39 (1.4)	92 (17.4)	9 (18.7)	140 (4.3)
Transferred out	42 (1.6)	7 (1.3)	1 (2.1)	50 (1.5)

TB = tuberculosis;

MDR = multidrug-resistant;

XDR = extensively drug-resistant.

**Table 5:** Comparison of treatment outcomes for hospitalized TB patients in the 309 Hospital stratified by treatment period (patients being treated before and after 2001).

	Treatment success	Poor treatment outcomes	OR (95%CI)	P
Before 2001				
Patients with non-MDR -TB				
New	27 (71.1)	11 (28.9)	1	
Retreatment	117 (84.8)	21 (15.2)	0.44 (0.19,1.02)	0.052
Patients with MDR-TB excluding XDR-TB				
New	6 (37.5)	10 (62.5)	1	
Retreatment	30 (30.0)	70 (70.0)	1.40 (0.47, 4.20)	0.547
Patients with XDR-TB				
New	1 (33.3)	2 (66.7)	1	
Retreatment	1 (7.7)	12 (92.3)	6.00 (0.26,140.05)	0.226
After 2001				
Patients with non-MDR -TB				
New	1088 (92.8)	85 (7.2)	1	
Retreatment	1218 (90.6)	127 (9.4)	1.34 (1.00,1.78)	0.048
Patients with MDR-TB excluding XDR-TB				
New	89 (59.3)	61 (40.7)	1	
Retreatment	157 (59.9)	105 (40.1)	0.98 (0.65, 1.47)	0.906
Patients with XDR-TB				
New	4 (33.3)	8 (66.7)	1	
Retreatment	8 (40.0)	12 (60.0)	0.75 (0.17,3.35)	0.706
Total				
Before 2001				
New	34 (59.6)	23 (40.4)	1	
Retreatment	148 (59.0)	103 (41.0)	1.03 (0.57, 1.85)	0.924
After 2001				
New	1181(88.5)	154 (11.5)	1	
Retreatment	1383 (85.0)	244(15.0)	1.35 (1.09, 1.68)	0.006

OR = odds ratio.

CI = confidence interval.

TB = tuberculosis;

MDR = multidrug-resistant;

XDR = extensively drug-resistant.

### Risk factors associated with poor treatment outcomes in MDR- and XDR-TB patients

Independent risk factors associated with poor treatment outcomes in MDR-TB patients were migrants (adjusted OR = 1.77, 95% CI, 1.06 to 2.96), smear-positivity at treatment onset (adjusted OR = 1.94, 95% CI, 1.13 to 3.34) and not receiving 3 or more potentially effective drugs (adjusted OR = 3.87, 95% CI, 2.53 to 5.91). Independent risk factors associated with poor treatment outcomes in XDR-TB patients were smear-positivity at treatment onset (adjusted OR = 10.42, 95% CI, 1.05 to 103.10) and not receiving 3 or more potentially effective drugs (adjusted OR = 14.90, 95% CI, 1.35 to 164.94) (Table 6 and Table S1).

**Table 6:** Univariate and multivariate logistic regress analysis of the association of potential risk factors with poor treatment outcomes in MDR- and XDR-TB patients*.

Potential risk factors	Patients with MDR-TB excluding XDR-TB				Patients with XDR-TB			
	Univariate analysis		Multivariate analysis		Univariate analysis		Multivariate analysis	
	OR (95% CI)	P	OR (95% CI)	P	OR (95% CI)	P	OR (95% CI)	P
Residence situation								
Beijing Resident	1		1		1		1	
Migrant	1.90 (1.33, 2.71)	<0.001	1.77 (1.06, 2.96)	0.030	4.32 (1.16,16.15)	0.030	4.58 (0.69, 30.33)	0.115
Living area								
Rural area	1		1		1			
Urban area	0.60 (0.42, 0.86)	0.005	0.75 (0.46,1.24)	0.265	0.50 (0.12, 2.15)	0.351		
Lower lung field TB	1.39 (0.98,1.97)	0.067	1.07 (0.72,1.60)	0.747	1.10 (0.29, 4.21)	0.891		
Sites of TB								
Extrapulmonary TB	1				1		1	
Pulmonary TB	1.91 (0.81,4.50)	0.140	2.06 (0.31,13.64)	0.454	5.50 (0.46, 66.32)	0.180	1.04 (0.05, 24.19)	0.981
Smear-positivity at treatment onset**	1.72 (1.06,2.80)	0.027	1.94 (1.13, 3.34)	0.016	6.72 (1.47, 30.76)	0.014	10.42 (1.05,103.10)	0.045
Radiological findings at onset								
Non-cavitary	1		1		1			
Cavitary disease	1.50 (1.06,2.12)	0.022	1.49 (0.99,2.24)	0.056	0.79 (0.22, 2.88)	0.725		
Diabetes mellitus	0.65 (0.37,1.14)	0.131	0.73 (0.38,1.43)	0.369	0.18 (0.02, 2.19)	0.180	0.20 (0.01,3.38)	0.263
Hypertension	0.32 (0.12,0.89)	0.029	0.63 (0.20,1.96)	0.423	1.73 (0.18,17.05)	0.637		
4 or more previous hospitalization for TB	0.55 (0.27,1.13)	0.103	0.53 (0.22,1.14)	0.101	0.00	0.999		
Resistance to 3 or more first-line drugs	1.52 (0.92,2.51)	0.105	1.06 (0.59,1.89)	0.852	0.76 (0.17,3.35)	0.714		
Not receiving 3 or more potentially effective drugs	2.77 (1.94,3.96)	<0.001	3.87 (2.53,5.91)	<0.001	8.57 (1.65, 44.43)	0.010	14.90 (1.35, 164.94)	0.028

TB = tuberculosis;

MDR = multidrug-resistant;

XDR = extensively drug-resistant.

OR = odds ratio.

CI = confidence interval.

*Variables included in the univariate analysis were shown in Table S1; All variables with a *P* value<0.2 in the univariate analysis were included in multivariate logistic regression analysis; 472 MDR-TB patients and 44 XDR-TB patients were included in multivariate logistic regression analysis. A *P* value of <0.05 was considered to be statistically significant.

**n = 477 for MDR-TB; n = 44 for XDR-TB.

### Risk factors associated with death in TB patients

Fifty of the 3270 enrolled TB patients died within two years following the start of the treatment. The median interval from anti-TB treatment to death was 7.3 months (IQR = 3.4–13.9). The 50 deaths occurred proportionally as follows: 31(1.2%) of 2694 patients with non-MDR-TB; 16 (3.0%) of 528 patients with MDR-TB excluding XDR-TB; and 3 (6.3%) of 48 patients with XDR-TB. In the Cox multivariate model, having chronic obstructive pulmonary disease (adjusted HR = 5.25, 95% CI, 2.60 to 10.62) and having hypertension (adjusted HR = 4.31, 95% CI, 1.90 to 9.78) were independent risk factors associated with death from any cause (Table 7 and Table S2).

**Table 7:** Cox proportional hazards regression model of factors associated with risk of death in all TB patients (n = 3270)*.

Variables	Univariate analysis		Multivariate analysis	
	HR (95% CI)	P	HR (95% CI)	P
Age				
0–14	1		1	
15–29	0.20 (0.03, 1.53)	0.121	0.22 (0.03, 1.74)	0.151
30–44	0.20 (0.03, 1.55)	0.123	0.16 (0.02, 1.25)	0.080
45–59	0.26 (0.03, 2.06)	0.204	0.25 (0.03, 2.02)	0.192
60–74	0.43 (0.06,3.35)	0.422	0.30 (0.04,2.40)	0.255
75–	0.45 (0.06, 3.66)	0.455	0.27 (0.03, 2.35)	0.236
Smear-positivity at treatment onset**	2.29 (1.03,5.08)	0.042	1.96 (0.86,4.44)	0.109
Cavitary disease	2.55 (1.42,4.58)	0.002	1.74 (0.95,3.19)	0.072
Chronic obstructive pulmonary disease	5.34 (2.74,10.44)	<0.001	5.25 (2.60,10.62)	<0.001
Hypertension	4.06 (1.97,8.36)	<0.001	4.31 (1.90,9.78)	<0.001
Not receiving 3 or more potentially effective drugs	1.76 (0.98,3.16)	0.058	1.30 (0.69,2.45)	0.413
MDR-TB	2.46 (1.36,4.46)	0.003	1.81 (0.96,3.41)	0.068
XDR-TB	4.36 (1.36,14.00)	0.013	2.20 (0.57,8.42)	0.251

TB = tuberculosis;

MDR = multidrug-resistant;

XDR = extensively drug-resistant.

HR = hazard ratio.

CI = confidence interval.

*Variables included in the univariate analysis were shown in Table S2; All variables with a *P* value<0.2 in the univariate analysis were included in multivariate Cox regression analysis; A *P* value of <0.05 was considered to be statistically significant.

**n = 2674.

## Discussion

The percentage of MDR-TB (20.2%) and XDR-TB (1.4%) among patients receiving inpatient treatment in our hospital from 1996 to 2009 was higher than the latest nationwide baseline survey for TB drug resistance (8.32% for MDR-TB and 0.68% for XDR-TB, respectively) [8]. The relatively higher prevalence of patients with MDR- and XDR-TB, and the overall high percentage of resistance to most of the anti-TB drugs tested could be explained by the fact that our study subjects were hospitalized TB patients, and were mainly comprised of long-term (52.6% had 4 or more years of TB disease), severe (54.4% had cavitary diseases, 73.0% had positive smear results, and 12.0% had Diabetes mellitus) and retreatment cases (57.4%).

A study from Taiwan reported the prevalence of MDR- and XDR-TB as being 5.7% (150/2625) and 0.4% (10/2625) respectively [26], and another study reported the prevalence of MDR- and XDR-TB as being 43.1% (261/605) and 5.5% (33/605) respectively among hospitalized TB patients in Georgia [27]. In our study, a high percentage of new (not previously treated) MDR-TB (31.4%, 166/528) and XDR-TB (31.3%, 15/48) cases were observed. A study conducted in Shanghai, another major city in China, also reported similar results. This latter study reported that more than half (59.4%, 104/175) of the patients with MDR- and XDR-TB were newly diagnosed and had no prior history of TB treatment. This suggests that there was a high ongoing transmission rate of MDR- and XDR-TB in major cities in China [15].

Discrepancies regarding the treatment success rates for MDR- and XDR-TB patients are reported in the literature. Treatment success rates have been shown to be associated with the degree of drug resistance and the presence or absence of HIV co-infection [19]. In the absence of HIV co-infection, the rates of treatment success in MDR- and XDR-TB reached 89% and 80% respectively among 125 TB patients with a definitive outcome in a study conducted in Germany [11]. Similar high treatment success rates were also observed, for example, in a recent study carried out in Heilongjiang province of China, the initial treatment success rates were 88.5% (100/113) and 73.4% (94/128) for newly diagnosed and retreated MDR-TB cases respectively [20]. A study in Peru also reported a high treatment success rate of 71% (10/14) for XDR-TB [12]. Relatively worse treatment outcomes were observed in some other studies. For example, the percentage of treatment success was 60.4% (142/235) for MDR-TB patients and 42.6% (23/54) for XDR-TB patients from a study in Estonia [14]. In a study conducted in Shanghai, only 54.9% (96/175) of the MDR-TB patients diagnosed in district TB hospitals in Shanghai were successfully treated [15]. In a study from South Africa, 49% (239/491) of the MDR-TB patients were cured or completed treatment [9]. Although overall treatment outcomes were reported to be substantially worse when HIV co-infection was also a consideration [17], a recent study from KwaZulu Natal, South Africa has reported that the number of deaths did not significantly differ in those patients with HIV infection (41%, 34/82) in comparison with non-HIV sufferers (30%, 28/92) [18]. In our study, the overall treatment success rates for MDR- and XDR-TB cases (53.4% for MDR-TB excluding XDR, and 29.2% for XDR-TB) were relatively low in comparison with studies from other cities in China and other countries in the world. In addition, the observation that the treatment outcome is less favorable in patients with XDR-TB than those with MDR-TB excluding XDR-TB as observed in our study and others is of concern. Because XDR-TB cases have even more limited choices of effective drugs available for treatment, it is highly unlikely that suitable treatment regimens involving at least three effective TB drugs can be designed in many cases [28].

We observed a significantly higher proportion of successful treatment in patients being treated after 2001 than before 2001. The improved treatment outcomes could be attributed to the fact that there had been no established standard guidelines for treating TB patients in the 309 Hospital until 2001, when the National Tuberculosis Programme (NTP) treatment guidelines were widely adopted in China. The improved treatment outcomes could also be linked to the overall success in TB control achieved in China due to increased government commitment and more intensive TB control measures such as covering the WHO Directly Observed Therapy Short-Course (DOTS) strategy nationwide, strengthening local public health facilities and national infrastructure [2]. Additionally, the data from our study also suggested that at least in this setting, and potentially in other similar settings with high drug resistance, DOTS programs are important, but it is not a measure sufficient enough to cure a sufficient number of TB cases with more complicated forms of TB such as MDR- and XDR-TB. The WHO guidelines recommend continuing therapy for a minimum of 18 months after culture conversion, and extending therapy to 24 months may be indicated in chronic cases with extensive pulmonary damage [22]. However, as a result of financial constraints, many patients with MDR- and XDR-TB in our study could not afford the cost of treatment and thus had much shorter treatment duration than would have been advised. In future, more efforts should be made to provide financial support for those MDR- and XDR-TB patients who cannot afford ideal individualised treatment regimens.

The proportion of defaulters among MDR-TB (92/528, 17.4%) and XDR-TB (9/48, 18.7%) patients in our study were close to that observed in Uzbekistan (12/87, 14% for MDR-TB) [16]. A higher proportion of defaulters were observed in a few other studies such as in South Africa (144/491, 29.0% for MDR-TB) [9] and South Korea (453/1407, 32.0% for MDR-TB) [13]. The high default rates observed in our study and others deserves important attention since all of the defaulted MDR- and XDR-TB patients without adequate treatment in our study had positive culture results and thus could potentially continue to be sources of transmission in their communities and threats to global public health [28]. The prognosis for the defaulted patients was suggested to be poor by a study reporting that of 47 (70.1%) MDR-TB patients who defaulted and were successfully traced, 25 (53.2%) had died [29]. Therefore, minimizing the rate of default is likely to not only help decrease ongoing transmission of MDR- and XDR-TB, but also reduce TB-related mortality.

Not receiving 3 or more potentially effective drugs was a risk factor significantly associated with poor treatment outcomes in both MDR-TB and XDR-TB patients in this study. Several studies suggested that extensive DST and the availability of second- and third-line drugs under inpatient management conditions permitted relatively high treatment success rates in MDR- and XDR-TB [11], [29], [30]. Currently, DST processing may take as long as several weeks in most clinics, by which time the patients could have already been treated with mono-therapy before the initiation of individualized treatment based on the DST results. Since many TB patients only receive in-patient treatment for less than 2 months due to financial restrictions, a proportion of new MDR- and XDR-TB cases will not be promptly diagnosed during these patients' hospitalization, affecting the overall TB treatment success rates. Therefore, rapid methods for early diagnosis of drug resistant-TB are urgently needed in order to achieve better treatment outcomes for MDR- and XDR-TB cases.

Migrants from other provinces and smear-positivity were also risk factors associated with poor treatment outcomes in patients with MDR-TB. Studies confirmed the migrant population contribution to the incidence of TB and the differences were reported among the 18 districts in Beijing [24], [31]. As the migrants posed great challenges on TB control, the Beijing municipal government has increased its efforts at addressing this issue, for example, by providing free anti-TB drugs for its migrant population since 2006. However, the government's free treatment policy is only able to cover a small percentage of the patients' total costs for TB treatment, as most migrant patients who contract TB are no longer able to work, this is not a suitable financial solution. Thus, more effort should be made to improve the social welfare, to include greater financial subsidies to TB treatment costs for the poor migrant TB patients. This is likely to achieve better treatment outcomes. Special attention may also beneficially be paid to migrant patients, with view to improve the treatment adherence in Beijing.

Regarding smear-positivity, our observation that having positive AFB smears at the start of treatment is independently associated with poor treatment outcomes in MDR-TB cases is in accordance with the findings from other studies [14], [32]. This may be explained by the fact that smear-positive patients often have more severe disease and a longer delay before the initiation of the treatment.

Although several studies have suggested an association between resistance to fluoroquinolones and poor treatment outcomes in MDR-TB patients [14], [32]–[34], this was not observed in our study. In addition, we also did not find any significant association between poor treatment outcomes and a few other variables previously identified to be risk factors for poor treatment outcomes in MDR- and XDR-TB patients, including previous TB treatment [14], alcohol abuse [32], and being male [32] for poor outcomes in patients with MDR-TB. Patients that were urban residents were at greater risk of contracting XDR-TB [14]. This disparity among different studies could be explained by the difference in study design and setting.

The observed mortality rates for patients with MDR- and XDR-TB (3.0% and 6.3%, respectively) in our study are relatively lower than those reported by a few other studies evaluating early treatment outcomes of patients with MDR- and XDR-TB. For example, in a study conducted in Karakalpakstan, Uzbekistan in 2003–2005, 15% of the 87 MDR-TB patients who were started on treatment during that period died [16]. In another study analysing early treatment outcomes of patients with XDR-TB in South Africa, 62 (36%) of the 174 patients with XDR-TB died, and the proportion of deaths was not significantly different in patients with or without HIV infection (41%, 34/82 versus 30%, 28/92) [18]. The difference in mortality rates in patients who suffered from MDR- and XDR-TB as reported in the above studies may be explained by clinical condition of the TB patients, the treatment regimens and protocols they received as well as the rates of defaulters in those studies.

The Cox proportional hazards model analysis that was performed showed that having chronic obstructive pulmonary disease and also hypertension were independent risk factors associated with death among TB patients, and having MDR-TB and cavitory disease were contributory to be risk factors associated with death among TB patients. Those findings further confirmed previous observation that pre-existing anti-TB drugs resistance and poor clinical condition of TB patients are common risk factors associated with death in TB patients [10], [16], [18].

Our study has several limitations. Firstly, some data on smoking (4.6%), alcohol abuse (3.6%), lower lung field TB (7.5%), AFB smear results at treatment onset (18.2%) and family history (2.0%) were not available at the time the study was performed. Secondly, this study was limited by the relatively small number of XDR-TB cases which were included in the analysis. It is likely that a larger patient cohort would have shown a more significant association between poor treatment outcomes and certain variables, including being migrants, having underlying diseases such as chronic obstructive pulmonary disease and having abnormal liver function for the XDR-TB patients. Thirdly, a relatively large proportion of patients without available medical records might have introduced a selection bias in our study. Those medical records were either borrowed by medical staff for treatment purposes or were taken away by those patients who were defaulted or were transferred out. Since the prognosis for defaulted patients was suggested to be poor [29], the fact that there were 188 of the 716 MDR-TB patients' medical records missing in our analysis could lead to under-estimation of poor treatment outcomes. We only had monitoring data for further deaths in the studied patients at 2 years after starting treatment, and no data was available on TB relapse and the long-term outcomes such as death rates in >2 years following the TB treatment. However, the long-term outcomes may be much worse than the initial treatment outcomes which were observed in our study, based on the data from the study in Heilongjiang province of China [20], which reported that the overall recurrence rates among new and retreatment MDR-TB cases were 46% and 66% respectively, and the overall death rates among new and retreatment MDR-TB cases were 25% and 46% respectively 4 years following treatment, even though much higher initial treatment success rates were observed in their study than that in our study. Long-term follow up of the treatment outcomes for TB patients, especially for MDR- and XDR-TB patients, is important. However, such follow-up study is difficult to conduct, as only slightly more than half of patients were included in the follow-up study in the Heilongjiang study, which may have introduced bias into the estimation of the recurrence and death rates, there is also the possibility of unreliable conclusions being drawn from any data that is collected.

Despite these limitations, our study presents some useful implications for basic research and clinical case management of patients with MDR- and XDR-TB. As MDR- and XDR-TB usually follow a much longer treatment course involving more expensive, less effective and more toxic second- or third-line anti-TB drugs, effort should be made to develop new and affordable drugs with better efficacy and fewer side effects. For laboratory diagnosis, economical and rapid molecular methods for the early diagnosis of TB and the determination of drug resistance profiles should be developed and applied to both new and retreated TB cases to provide timely information with which appropriate individualised regimens for MDR- and XDR-TB cases may be developed. Clinically, more effort should be made to minimise the number of defaulted patients. Greater measures should be taken to provide adequate treatment for vulnerable populations such as poor migrant TB patients and those who have serious underlying diseases such as chronic obstructive pulmonary disease and hypertension. In addition, more intensive efforts should be made to manage MDR- and XDR-TB cases more effectively to improve their treatment outcomes of all patients and hence minimise further development of so-called totally drug-resistant TB cases [35].

## Supporting Information

Table S1 **Univariate logistic regress analysis of the association of potential risk factors with poor treatment outcomes in MDR- and XDR-TB patients.**
(DOC)

Table S2 **Cox proportional hazards regression model of factors associated with risk of death in all TB patients (n = 3270).**
(DOC)
