# Supplementary material for: Characteristics and Treatment Outcomes of Patients with MDR and XDR Tuberculosis in a TB Referral Hospital in Beijing: A 13-Year Experience
Source: PLoS One. 2011 Apr 29;6(4):e19399. doi: 10.1371/journal.pone.0019399 (PMC3084844; doi:10.1371/journal.pone.0019399)
Supplement: Table S1 — Univariate logistic regress analysis of the association of potential risk factors with poor treatment outcomes in MDR- and XDR-TB patients. (DOC) [file pone.0019399.s001.doc]

**Table S1. Univariate logistic regress analysis of the association of potential risk factors with poor treatment outcomes in MDR- and XDR-TB patients.**

| **Variables** | **Patients with MDR-TB excluding XDR-TB** | | | **Patients with XDR-TB** | | |
| --- | --- | --- | --- | --- | --- | --- |
|  | **Poor treatment outcomes** | **Univariate analysis** | | **Poor treatment outcomes** | **Univariate analysis** | |
|  | **n=246 (46.6%)** | **OR (95% CI)** | **P** | **n=34 (70.8%)** | **OR (95% CI)** | **P** |
| Gender |  |  |  |  |  |  |
| Male | 156/345 (45.2) | 1 |  | 26/36 (72.2) | 1 |  |
| Female | 90/183 (49.2) | 1.17 (0.82, 1.68) | 0.385 | 8/12 (66.7) | 0.77 (0.19, 3.13) | 0.714 |
| Age |  |  |  |  |  |  |
| 0-14 | 2/4 (50.0) | 1 |  | 1/2 (50.0) | 1 |  |
| 15-29 | 80/149 (53.7) | 1.16 (0.16, 8.45) | 0.884 | 10/16 (62.5) | 1.67 (0.09, 31.87) | 0.734 |
| 30-44 | 82/171 (48.0) | 0.92 (0.13, 6.69) | 0.935 | 13/17 (76.5) | 3.25 (0.16, 64.61) | 0.440 |
| 45-59 | 36/79 (45.6) | 0.84 (0.11, 6.24) | 0.862 | 2/2 (100.0) | 0.00 | 0.999 |
| 60-74 | 34/87 (39.1) | 0.64 (0.09, 4.77) | 0.665 | 6/8 (75.0) | 3.00 (0.12, 73.64) | 0.501 |
| 75- | 12/38 (31.6) | 0.46 (0.06, 3.68) | 0.465 | 2/3 (66.7) | 2.00 (0.05, 78.25) | 0.711 |
| Marital status |  |  |  |  |  |  |
| Married | 192/413 (46.5) | 1 |  | 23/30 (76.7) | 1 |  |
| Single | 54/115 (47.0) | 0.98 (0.65, 1.49) | 0.929 | 11/18 (61.1) | 2.09 (0.59, 7.45) | 0.255 |
| Residence situation |  |  |  |  |  |  |
| Beijing Resident | 78/210 (37.1) | 1 |  | 10/19 (52.6) | 1 |  |
| Migrant | 168/318 (52.8) | 1.90 (1.33,2.71) | <0.001 | 24/29 (82.8) | 4.32 (1.16,16.15) | 0.030 |
| Living area |  |  |  |  |  |  |
| Rural area | 107/196 (54.6) | 1 |  | 12/15 (80.0) | 1 |  |
| Urban area | 139/332 (41.9) | 0.60 (0.42,0.86) | 0.005 | 22/33 (66.7) | 0.50 (0.12,2.15) | 0.351 |
| Ethnicity |  |  |  |  |  |  |
| The largest group (Han) | 235/508 (46.3) | 1 |  | 32/45 (71.1) | 1 |  |
| Ethnic groups | 11/20 (55.0) | 1.42 (0.58,3.49) | 0.444 | 2/3 (66.7) | 0.81 (0.07,9.76) | 0.870 |
| Smoking, yes * | 36/78 (46.2) | 0.98 (0.60,1.59) | 0.936 | 4/5 (80.0) | 1.92 (0.19,19.09) | 0.578 |
| Alcohol abuse, yes ** | 11/20 (55.0) | 1.46 (0.59,3.59) | 0.410 | 2/2 (100.0) | 0.00 | 0.999 |
| Sites of TB |  |  |  |  |  |  |
| Extrapulmonary TB | 8/25 (32.0) | 1 |  | 1/3 (33.3) | 1 |  |
| Pulmonary TB | 238/503 (47.3) | 1.91 (0.81,4.50) | 0.140 | 33/45 (73.3) | 5.50 (0.46,66.32) | 0.180 |
| Lower lung field TB *** | 134/262 (51.1) | 1.39 (0.98,1.97) | 0.067 | 20/27 (74.1) | 1.10 (0.29,4.21) | 0.891 |
| TB history |  |  |  |  |  |  |
| New | 76/166 (45.8) | 1 |  | 9/15 (60.0) | 1 |  |
| Retreatment | 170/362 (47.0) | 1.05 (0.73,1.52) | 0.801 | 25/33 (75.8) | 2.08 (0.57, 7.68) | 0.270 |
| Smear-positivity at treatment onset **** | 195/392 (49.7) | 1.72 (1.06,2.80) | 0.027 | 28/33 (84.8) | 6.72 (1.47, 30.76) | 0.014 |
| Radiological findings at onset |  |  |  |  |  |  |
| Non-cavitary | 96/234 (41.0) | 1 |  | 14/19 (73.7) | 1 |  |
| Cavitary disease | 150/294 (51.0) | 1.50 (1.06,2.12) | 0.022 | 20/29 (69.0) | 0.79 (0.22, 2.88) | 0.725 |
| Family history of TB, yes***** | 16/31 (51.6) | 1.27 (0.61,2.63) | 0.519 | 1/2 (50.0) | 0.41 (0.02, 6.99) | 0.535 |
| Underlying diseases |  |  |  |  |  |  |
| Diabetes mellitus | 22/59 (37.3) | 0.65 (0.37,1.14) | 0.131 | 1/3 (33.3) | 0.18 (0.02, 2.19) | 0.180 |
| Chronic obstructive pulmonary disease | 12/22 (54.5) | 1.40 (0.59,3.29) | 0.447 | 8/9 (88.9) | 4.00 (0.45,35.49) | 0.213 |
| A[bnormal](http://www.iciba.com/abnormal/) liver function | 2/8 (25.0) | 0.38 (0.08,1.89) | 0.235 | 8/11 (72.7) | 1.13 (0.25, 5.07) | 0.875 |
| Hepatitis | 7/19 (36.8) | 0.66 (0.26,1.70) | 0.389 | 1/3 (33.3) | 0.18 (0.02,2.19) | 0.180 |
| Malignancy | 0/1 (0.0) | 0.00 | 1.000 | 0/0 |  |  |
| Hypertension | 5/22 (22.7) | 0.32 (0.12,0.89) | 0.029 | 4/5 (80.0) | 1.73 (0.18,17.05) | 0.637 |
| 4 or more previous hospitalization for TB | 12/36 (33.3) | 0.55 (0.27,1.13) | 0.103 | 4/4 (100.0) | 0.00 | 0.999 |
| 4 or more years of TB disease | 189/406 (46.6) | 0.99 (0.66,1.49) | 0.974 | 31/42 (73.8) | 2.82 (0.49,16.09) | 0.244 |
| Any resistance to ofloxacin | 31/66 (47.0) | 1.02 (0.61,1.71) | 0.947 | 34/48 (70.8) |  |  |
| Any resistance to kanamycin | 59/137 (43.1) | 0.83 (0.56,1.22) | 0.337 | 34/48 (70.8) |  |  |
| Any resistance to para-aminosalicylic acid | 57/129 (44.2) | 0.88 (0.59,1.31) | 0.529 | 11/14 (78.6) | 1.75 (0.41,7.59) | 0.449 |
| Resistance to 3 or more first-line drugs | 218/454 (48.0) | 1.52 (0.92,2.51) | 0.105 | 25/36 (69.4) | 0.76 (0.17,3.35) | 0.714 |
| Resistance to 2 or more second-line drugs | 30/66 (45.5) | 0.95 (0.57,1.59) | 0.843 | 34/48 (70.8) |  |  |
| Resistance to 5 or more any drugs | 116/247 (47.0) | 1.03 (0.73,1.45) | 0.872 | 31/43 (72.1) | 1.72 (0.26, 11.62) | 0.577 |
| Not receiving 3 or more potentially effective drugs | 137/225 (60.9) | 2.77 (1.94,3.96) | <0.001 | 20/22 (90.9) | 8.57 (1.65, 44.43) | 0.010 |

TB = tuberculosis;

MDR = multidrug-resistant;

XDR = extensively drug-resistant.

OR = odds ratio.

CI = confidence interval.

*n = 496 for MDR-TB; n= 42 for XDR-TB

** n = 496 for MDR-TB; n= 44 for XDR-TB

*** n = 504 for MDR-TB; n= 45 for XDR-TB

**** n = 477 for MDR-TB; n= 44 for XDR-TB

***** n = 502 for MDR-TB; n= 47 for XDR-TB

All variables with a *P* value < 0.2 in the univariate analysis were considered for the multivariate logistic regression model. A *P* value of <0.05 was considered to be statistically significant.
