# Supplementary material for: Characteristics and Treatment Outcomes of Patients with MDR and XDR Tuberculosis in a TB Referral Hospital in Beijing: A 13-Year Experience
Source: PLoS One. 2011 Apr 29;6(4):e19399. doi: 10.1371/journal.pone.0019399 (PMC3084844; doi:10.1371/journal.pone.0019399)
Supplement: Table S2 — Cox proportional hazards regression model of factors associated with risk of death in all TB patients (n = 3270). (DOC) [file pone.0019399.s002.doc]

**Table S2. Cox proportional hazards regression model of factors associated with risk of death in all TB patients (n=3270).**

| **Variables** | **Death** | **Univariate analysis** | | **Multivariate analysis** | |
| --- | --- | --- | --- | --- | --- |
|  | **n=50 (1.5%)** | **HR (95% CI)** | **P** | **HR (95% CI)** | **P** |
| Gender |  |  |  |  |  |
| Male | 32/2292 (1.4) | 1 |  |  |  |
| Female | 18/978 (1.8) | 1.32 (0.74, 2.35) | 0.346 |  |  |
| Age |  |  |  |  |  |
| 0-14 | 1/18 (5.6) | 1 |  | 1 |  |
| 15-29 | 11/974 (1.1) | 0.20 (0.03, 1.53) | 0.121 | 0.22 (0.03, 1.74) | 0.151 |
| 30-44 | 10/884 (1.1) | 0.20 (0.03, 1.55) | 0.123 | 0.16 (0.02, 1.25) | 0.080 |
| 45-59 | 10/668 (1.5) | 0.26 (0.03, 2.06) | 0.204 | 0.25 (0.03, 2.02) | 0.192 |
| 60-74 | 11/451 (2.4) | 0.43 (0.06, 3.35) | 0.422 | 0.30 (0.04, 2.40) | 0.255 |
| 75- | 7/275 (2.5) | 0.45 (0.06, 3.66) | 0.455 | 0.27 (0.03, 2.35) | 0.236 |
| Marital status |  |  |  |  |  |
| Married | 35/2443 (1.4) | 1 |  |  |  |
| Single | 15/827 (1.8) | 0.79 (0.43, 1.45) | 0.443 |  |  |
| Residence situation |  |  |  |  |  |
| Beijing Resident | 20/1421 (1.4) | 1 |  |  |  |
| Migrant | 30/1849 (1.6) | 1.15 (0.65,2.03) | 0.628 |  |  |
| Living area |  |  |  |  |  |
| Rural area | 18/1172 (1.5) | 1 |  |  |  |
| Urban area | 32/2098 (1.5) | 0.99 (0.56,1.77) | 0.982 |  |  |
| Ethnicity |  |  |  |  |  |
| The largest group (Han) | 48/3153 (1.5) | 1 |  |  |  |
| Ethnic groups | 2/117 (1.7) | 1.13 (0.27,4.65) | 0.867 |  |  |
| Smoking, yes * | 6/526 (1.1) | 0.72 (0.31,1.69) | 0.450 |  |  |
| Alcohol abuse, yes ** | 4/142 (2.8) | 1.94 (0.70,5.38) | 0.206 |  |  |
| Sites of TB |  |  |  |  |  |
| Extrapulmonary TB | 2/256 (0.8) | 1 |  |  |  |
| Pulmonary TB | 48/3014 (1.6) | 2.04 (0.50,8.40) | 0.323 |  |  |
| Lower lung field TB *** | 22/1575 (1.4) | 0.75 (0.43,1.31) | 0.313 |  |  |
| TB history |  |  |  |  |  |
| New | 18/1392 (1.3) | 1 |  |  |  |
| Retreatment | 32/1878 (1.7) | 1.32 (0.74,2.35) | 0.347 |  |  |
| Smear-positivity at treatment onset **** | 43/1951 (2.2) | 2.29 (1.03,5.08) | 0.042 | 1.96 (0.86,4.44) | 0.109 |
| Radiological findings at onset |  |  |  |  |  |
| Non-cavitary | 11/1492 (0.7) | 1 |  | 1 |  |
| Cavitary disease | 39/1778 (2.2) | 2.55 (1.42,4.58) | 0.002 | 1.74 (0.95,3.19) | 0.072 |
| Family history of TB, yes***** | 1/133 (0.8) | 0.47 (0.07, 3.40) | 0.455 |  |  |
| Underlying diseases |  |  |  |  |  |
| Diabetes mellitus | 7/393 (1.8) | 1.19 (0.54,2.65) | 0.668 |  |  |
| Chronic obstructive pulmonary disease | 11/168 (6.5) | 5.34 (2.74,10.44) | <0.001 | 5.25 (2.60,10.62) | <0.001 |
| A[bnormal](http://www.iciba.com/abnormal/) liver function | 5/190 (2.6) | 1.81 (0.72,4.56) | 0.209 |  |  |
| Hepatitis | 2/112 (1.8) | 1.18 (0.29,4.84) | 0.823 |  |  |
| Malignancy | 1/25 (4.0) | 2.69 (0.37,19.46) | 0.328 |  |  |
| Hypertension | 9/172 (5.2) | 4.06 (1.97,8.36) | <0.001 | 4.31 (1.90,9.78) | <0.001 |
| 4 or more previous hospitalization for TB | 4/175 (2.3) | 1.53 (0.55,4.25) | 0.415 |  |  |
| 4 or more years of TB disease | 23/1720 (1.3) | 0.77 (0.44,1.34) | 0.347 |  |  |
| Any resistance to ofloxacin | 7/314 (2.2) | 1.38(0.71,2.70) | 0.342 |  |  |
| Any resistance to kanamycin | 9/534 (1.7) | 1.05 (0.57,1.92) | 0.883 |  |  |
| Any resistance to para-aminosalicylic acid | 9/793 (1.1) | 0.68 (0.33,1.40) | 0.299 |  |  |
| Resistance to 3 or more first-line drugs | 20/1057 (1.9) | 1.40 (0.80,2.47) | 0.243 |  |  |
| Resistance to 2 or more second-line drugs | 9/636 (1.4) | 0.91 (0.44,1.87) | 0.791 |  |  |
| Resistance to 5 or more any drugs | 14/919 (1.5) | 1.00 (0.54,1.85) | 0.988 |  |  |
| Not receiving 3 or more potentially effective drugs | 17/744 (2.3) | 1.76 (0.98,3.16) | 0.058 | 1.30 (0.69,2.45) | 0.413 |
| MDR-TB | 16/528 (3.0) | 2.46 (1.36,4.46) | 0.003 | 1.81 (0.96,3.41) | 0.068 |
| XDR-TB | 3/48 (6.3) | 4.36 (1.36,14.00) | 0.013 | 2.20 (0.57,8.42) | 0.251 |

TB = tuberculosis;

MDR = multidrug-resistant;

XDR = extensively drug-resistant.

HR = hazard ratio.

CI = confidence interval.

*n = 3119

** n = 3151

*** n = 3024

**** n = 2674

***** n = 3205

All variables with a *P* value < 0.2 in the univariate analysis were considered for the multivariate Cox regression model. A *P* value of <0.05 was considered to be statistically significant.
